# Evaluation of Titanium Particles, TNF-α, and Caspase-3 Concentrations in Patients with Bones Fixations of the Maxilla and Mandibule

**DOI:** 10.3390/ijms26052316

**Published:** 2025-03-05

**Authors:** Bożena Antonowicz, Żaneta Anna Mierzejewska, Jan Borys, Mateusz Maciejczyk, Sławomir Prokopiuk, Halina Car

**Affiliations:** 1 Department of Dental Surgery, Medical University of Bialystok, 15-276 Bialystok, Poland; 2 Institute of Biomedical Engineering, Faculty of Mechanical Department, Bialystok University of Technology, 15-351 Bialystok, Poland; 3 Department of Maxillofacial and Plastic Surgery, Medical University of Bialystok, 15-276 Bialystok, Poland; jan.borys@umb.edu.pl; 4 Department of Hygiene, Epidemiology and Ergonomics, Medical University of Bialystok, 15-222 Bialystok, Poland; m.maciejczyk@umb.pl; 5 Faculty of Health Sciences, University of Lomza, 18-400 Lomza, Poland; sprokopiuk@al.edu.pl; 6 Department of Experimental Pharmacology, Medical University of Bialystok, 15-295 Bialystok, Poland; hcar@umb.edu.pl; 7 Department of Clinical Pharmacology, Medical University of Bialystok, 15-274 Bialystok, Poland

**Keywords:** TNF-α, caspase-3, titanium implants, jawbone fixations, miniplates and screws

## Abstract

The aim of the study was to evaluate the effect of titanium implants (Ti6Al4V) on the surrounding tissues by analyzing the concentration of titanium particles, TNF-α, and caspase-3 in patients treated for jaw fractures and dentofacial deformities. The research material consisted of peri-implant tissues: fragments of periosteum adhering to a titanium miniplate and blood serum collected from 42 patients treated for mandibular fractures (Group I), and dentofacial deformities (Group II) who underwent bimaxillary osteotomy. The control group consisted of 24 generally healthy patients before bimaxillary osteotomy. The concentrations of selected cytokines, caspase-3, TNF-α in blood serum, and homogenized tissues, were determined using the immunoenzymatic method (ELISA). The concentration of titanium particles was assessed using a scanning electron microscope equipped with an X-ray microanalyzer. A significant increase in the concentration of titanium, caspase-3, and TNF-α was observed in serum and periosteum in all patients who underwent bone fixation. Increased TNF-α levels indicate an intense immune response, which may lead to the degradation of peri-implant tissues and bone resorption around the miniplates and screws, while an increase in caspase-3 levels suggests that cells surrounding the implants are destroyed in response to inflammatory stress or damage induced by the presence of titanium particles.

## 1. Introduction

The last decades of the 20th century brought significant changes in the treatment of osteosynthesis in jaw fragments. They were the result of technological progress, a better understanding of the biomechanics of the facial skeleton, and the development of surgical techniques and materials used in medicine [1]. A natural consequence of this progress was the displacement of conservative methods of bone-fragment fixation and their replacement with surgical ones [2,3,4]. Currently, the standard in maxillofacial surgery is open reduction and internal fixation using plates and screws [5]. This method ensures a stable connection of bone fragments and allows for quick and precise healing with minimal risk of complications, the restoration of physiological functions of the stomatognathic system, and faster rehabilitation of patients, as well as the possibility of shortening the time of intermaxillary immobilization, which can be burdensome for patients [6].

Despite the undoubted advantages of plate osteosynthesis, in which the use of titanium mini-plates and screws has become the gold standard in the fixation of bone fragments of the facial skeleton, this method is not free from imperfections. The use of rigid jawbone fixation systems often requires their removal, and, therefore, another surgery, mainly due to the occurrence of local complications and healing problems, the inhibition of bone growth in children and adolescents, the aseptic loosening of plates and their migration, and the hypersensitivity of the face to thermal stimuli and image distortions in MRI and CT examinations [7,8,9,10].

The most commonly used material for the production of plates and screws is titanium alloys, which have a number of unique properties: they are well tolerated by the body, do not cause allergic reactions, and a protective layer of titanium oxide is formed on their surface, which means that these alloys do not react with body fluids or tissues, demonstrating high corrosion resistance. In addition, the modulus of elasticity of titanium alloys is closer to bone than other materials, which reduces the risk of stress shielding [11,12,13,14,15]. Although titanium is still considered a biocompatible material, current studies show that its use in implants can lead to long-term problems, especially in the case of contact with tissues with a high degree of mechanical load [16,17].

Metallosis is a phenomenon of metal particle deposition in tissues surrounding implants. An analysis of literature sources has shown that this problem was known earlier, as well as in cases of jawbone fixation [18,19,20]. In the human body, the degradation of titanium or its alloys may occur as a result of a violation of the structural integrity of the implant due to friction (fretting) and the accompanying electrochemical corrosion process [21,22]. Fretting is a type of friction occurring in conditions of micro-movements of elements constituting systems stabilizing bone fragments. Fretting usually leads to the intensification of tribological wear processes, abrasion, adhesion, formation of galvanic cells, and fatigue degradation of materials from which jawbone fixations are made. In this case of bone fixations, the passive (protective) layer is damaged, and the presence of hard wear particles in the friction zone, acting as an abrasive, intensifies secondary wear [22,23,24]. In vitro studies show that fretting corrosion in fixation plates is a primary corrosive process initiated by micro-movements in the areas of these fixations, which are intensified by the presence of a body-fluid environment [25,26].

**Figure 1:**
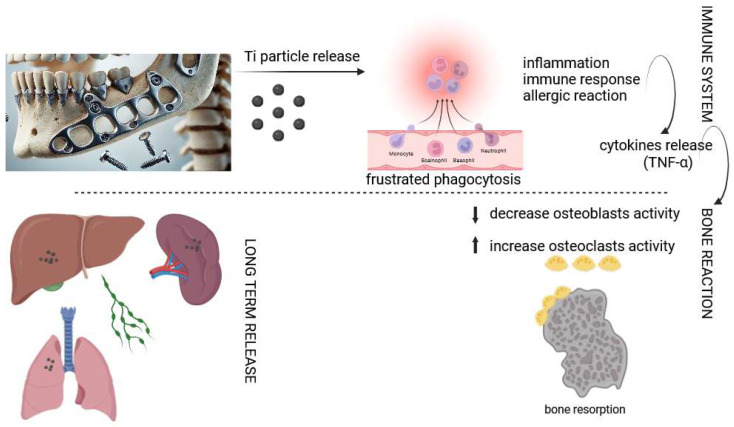
Schematic illustration depicting the initial effects caused by ions/particles released from dental implants into the surrounding tissues: immune response and bone resorption; migration or accumulation of ions/particles (BioRender.com, accessed on 25 January 2025).

The effects of metallosis can be observed in the tissues surrounding the metal bone fixations during their removal. The premise for undertaking the study was clinical observations and literature reports on excessive periosteal bone layering and discoloration of both bone and periosteum around the removed fixations, found in all operated patients, which is confirmed by previous research [27,28]. Moreover, as the literature data show, titanium particles causing discoloration were present not only in the peri-implant tissues but also in other organs: the lungs, spleen, liver, and lymph nodes [29,30]; Figure 1.

Titanium particles accumulating in the surrounding tissues, especially in soft tissue or bone, may cause the disruption of regenerative processes, an immunoinflammatory reaction of the body to a foreign body, bone resorption, and even chronic inflammation occurring in the area of bone connections, which may appear in patients in the form of recurrent edema, purulent fistulas in the oral cavity, and changes on the skin of the face and neck [22,24,26]. In clinical practice, the authors observed such symptoms in patients at various times after surgical treatment of jaw fractures or dentofacial deformities—from about a year to even 12 years after the procedure. The occurrence of edema and fistula was not caused by reasons other than the presence of fixation; odontogenic inflammation, the pressure of the denture base on the mucosa covering the mini-plates, gingival injuries, etc., were excluded.

Markers indicating the inflammatory process occurring in the body include TNF-α (tumor necrosis factor-alpha) and caspase-3. TNF-α is a pro-inflammatory cytokine that is released in response to various stimuli, including products of titanium implant degradation. It plays a central role in the inflammatory cascade, promoting the activation of immune cells such as macrophages and neutrophils. TNF-α initiates a signalling pathway that leads to the production of other inflammatory mediators, interleukins and chemokines, which additionally enhance the inflammatory response. Excessive expression of TNF-α can lead to chronic inflammation and damage to peri-implant tissues [31,32,33]. Caspase-3, in turn, is a key executive enzyme in the apoptosis pathway. Apoptosis, or programmed cell death, is an important process in maintaining tissue homeostasis. In the context of implant degradation, caspase-3 may be activated in response to cellular stress induced by titanium alloy particles. This enzyme mediates the breakdown of cellular components and leads to the death of affected cells, contributing to tissue remodeling and the development of inflammation. The interaction between TNF-α and caspase-3 in the inflammatory response underscores the complexity of tissue responses to titanium implants. Both markers serve as key indicators of the host response to implant degradation and may impact osseointegration and the long-term stability of the fixation. Elevated levels of these markers may signal ongoing inflammation and cellular stress, potentially leading to implant loosening and failure of the reconstructive procedure [34,35].

To summarize the above, the aim of this study was to determine the effect of titanium implants on the surrounding tissues by analyzing the metallosis process and the concentration of TNF-α and caspase-3 in patients treated for mandibular fractures and patients with dentofacial deformities who underwent titanium fixations.

## 2. Results

### 2.1. Analysis of TNF-α Concentration

**Figure 2:**
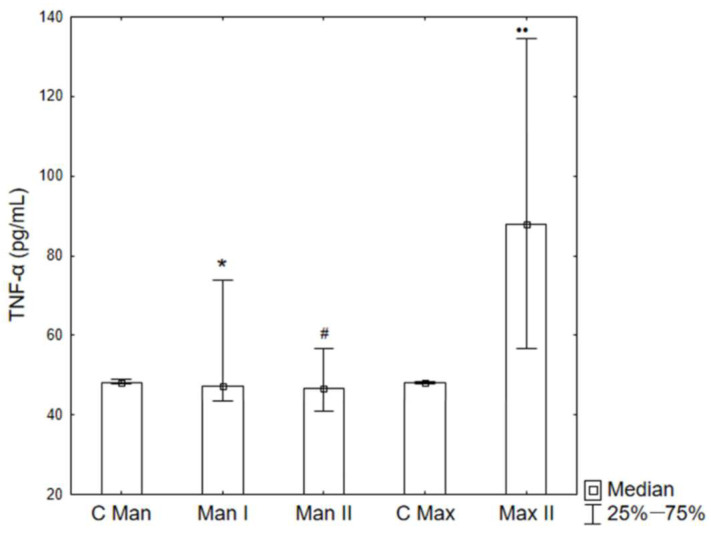
TNF-α concentrations in the periosteum. C Man—control group mandible, C Max—control group maxilla (before implantation), Man I—Group I mandible (fracture), Man II—Group II mandible, Max II—Group II maxilla (bimaxillary osteotomy). * *p* < 0.05 vs. C Max; # *p* < 0.05 vs. Man I; •• *p* < 0.05.

**Figure 3:**
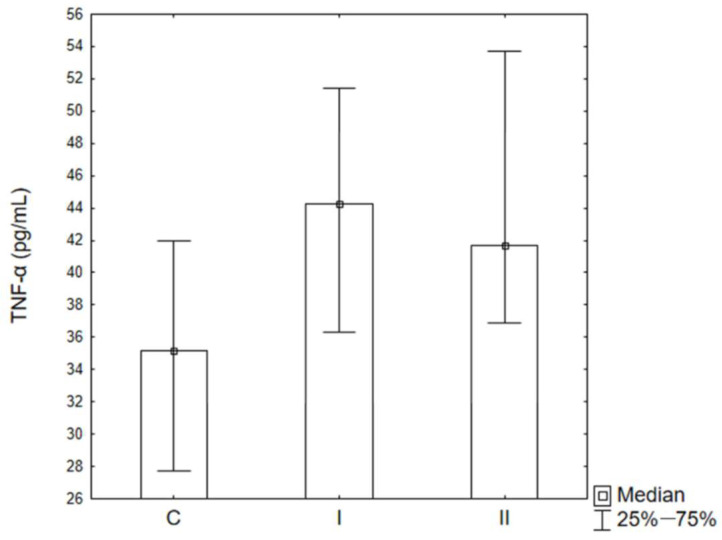
TNF-α concentrations in blood serum. C—control Group, I Group I (mandible fractures), II—Group II (bimaxillary osteotomy).

The concentration of TNF-α in the periosteum was examined before implantation of the titanium plate in Groups C Man and C Max, in patients after bimaxillary osteotomy (Man II and Max II), and in patients with a mandibular fracture (Man I). In the maxillary periosteum covering the bone fixation in patients from Group II (Max II), a statistically significant increase in TNF-α concentration was obtained compared to C Max, and it was significantly higher than in both Groups Man I and Man II. No statistically significant differences were observed between the concentrations of this cytokine in the periosteum collected from the mandible in Groups Man I and Man II (Figure 2). A tendency towards an increase in the concentration of TNF-α in the blood serum of the patients studied was observed in patients from both groups (Figure 3).

The statistically significant increase in TNF-α in the maxillary periosteum (Max II) compared to C Max suggests that bimaxillary osteotomy in this group induces a stronger inflammatory response than in the case of the mandible alone. The lack of significant differences in TNF-α concentration between the Man I and Man II groups may indicate a similar course of inflammatory and regenerative processes in the mandible, regardless of whether fracture or osteotomy occurred. However, when analyzing the difference in TNF-α concentration in the maxillary and mandibular periosteum in the Man II and Max II groups, it is necessary to emphasize the differences in the blood supply to the maxilla and mandible and the different load conditions. The maxilla, as a spongy bone, may show a stronger inflammatory response than the more compact mandible.

### 2.2. Analysis of Caspase-3 Concentration

**Figure 4:**
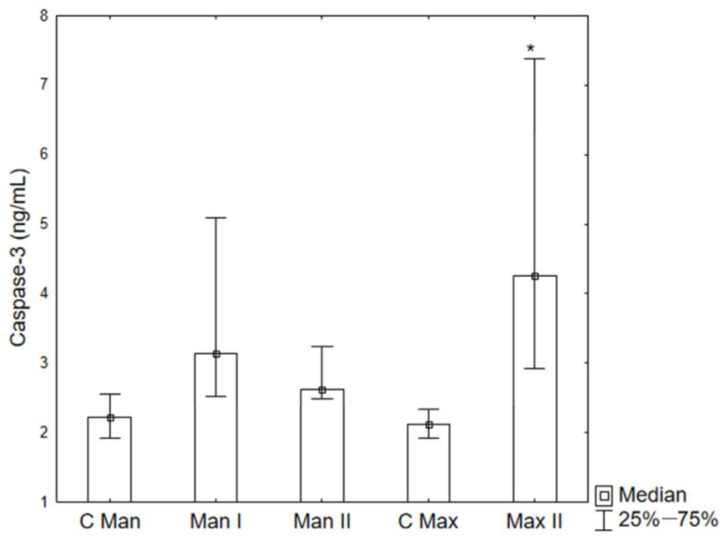
Caspase-3 concentrations in the periosteum C Man—control group mandible, C Max—control group maxilla (before implantation), Man I—Group I mandible (fracture), Man II—Group II mandible, Max II—Group II maxilla (bimaxillary osteotomy). * *p* < 0.05 vs. C Max.

**Figure 5:**
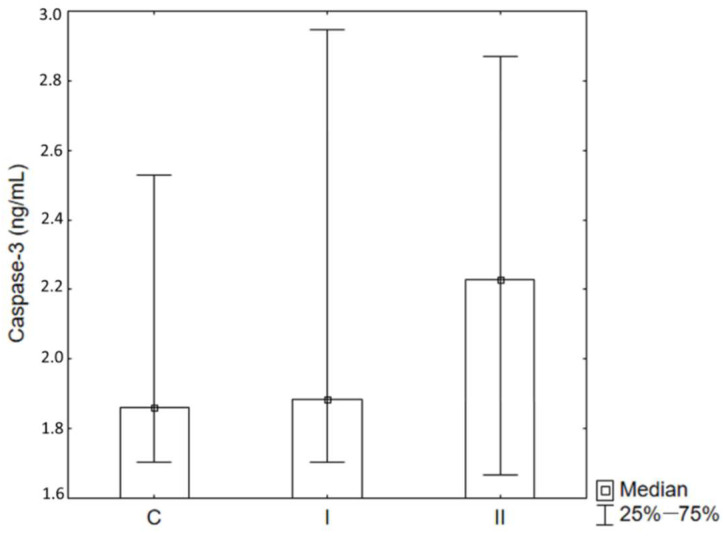
Caspase-3 concentrations in blood serum. C—Control Group I, Group I (mandible fractures), II—Group II (bimaxillary osteotomy).

The study indicates a statistically significant increase in the concentration of caspase-3 in the periosteum taken from the jaw of patients from Group II (Max II) compared to the control group (C Max). An upward trend was also observed in the concentration of caspase-3 in the periosteum taken from the mandible of patients from both Groups Man I and Man II (Figure 4). However, no significant changes were observed in the concentration of caspase-3 in the blood serum (Figure 5).

The increase in caspase-3 in the mandibular periosteum in patients after fracture (Man I) and osteotomy (Man II) indicates that trauma and surgery lead to the stimulation of apoptotic pathways, although this increase was not statistically significant. On the other hand, a statistically significant increase in caspase-3 concentration in the maxillary periosteum after bimaxillary osteotomy (Max II) indicates a more intensive activation of apoptotic processes in this location, which, in this case, may be the effect of the extent of surgical intervention, differences in vascularization, and a different structure of the maxillary bone tissue compared to the mandible.

The significantly increased concentration of caspase-3 in the serum of patients after bimaxillary osteotomy suggests that this procedure leads to a more intensive activation of apoptotic processes at the systemic level compared to the mandibular fracture. The lack of significant changes in the control group (C) and after the mandibular fracture (Man I) indicates that the fracture itself does not cause a systemic apoptotic response, and these processes remain limited to the site of injury. These results indicate that bimaxillary osteotomy is a more biologically burdensome procedure than the isolated mandibular fracture, which may be of clinical importance when monitoring healing and potential complications.

### 2.3. Analysis of Titanium Concentration

Before the bimaxillary osteotomy, the control group had no detectable levels of titanium in their blood, suggesting no previous exposure to this element in the context of surgical or implant procedures, whereas an examination of the periosteum after the removal of bone fixations confirmed the presence of titanium particles in the tissues of patients operated for mandibular fractures (I) and after bimaxillary osteotomy (II).

**Figure 6:**
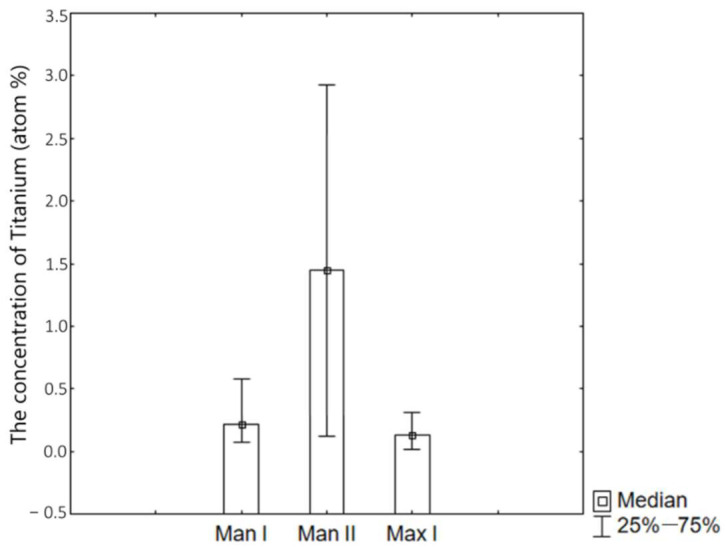
The concentration of titanium in the tested periosteum in Groups I and II.

**Figure 7:**
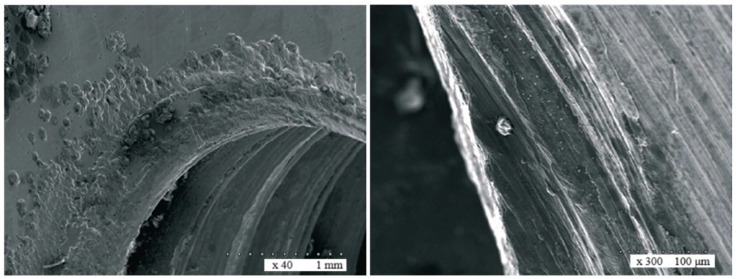
Sample image showing wear to the components of a plate fixation system, obtained from a scanning microscope.

**Figure 8:**
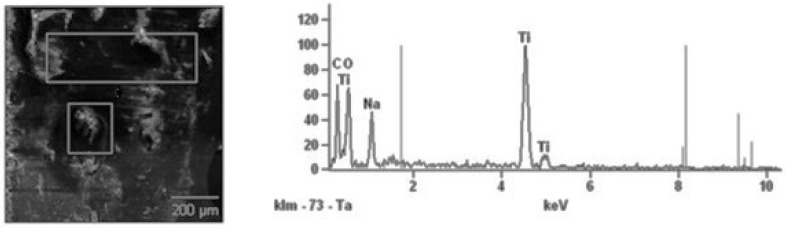
Image of a sample result of elemental analysis in the periosteum covering titanium jaw fixations obtained from a scanning microscope; corresponding chemical composition spectrum obtained by NSS X-ray microanalysis.

The highest concentrations of titanium in the periosteum were obtained in peri-implant tissues collected from the mandible of patients after bimaxillary osteotomy (Man II). The concentrations of titanium particles in tissues collected from the mandible of patients from the Man I group and from the maxilla of patients from the Max II group were at a comparable level (Figure 6, Figure 7 and Figure 8). An analysis of titanium concentration in blood plasma showed a significant increase in its level in all patients, regardless of the study group. In Group I, these values averaged 5.2 ± 1.1 ng/mL, while in Group II, they were higher and ranged around 8.6 ± 1.3 ng/mL. The process of osteointegration and bone remodeling, as well as complex conditions of mechanical loads acting around implants, favor their gradual wear and the penetration of microparticles into the periosteum. The lack of detection of titanium particles in the tissues of patients from the control group confirms that their source is titanium implants used in surgical procedures. This is important from the point of view of the biocompatibility of the material. Although titanium is considered to be well tolerated by the body, its microparticles can potentially affect the local inflammatory response and regenerative processes.

## 3. Discussion

Metallic prostheses, fixations, and anchor systems in the bone are widely used across the world in maxillofacial surgeries, orthopedics, and stomatology. Titanium (Ti) in pure form is highly reactive. Therefore, its perceived biocompatibility is associated with the presence of a highly passive layer (4 nm thick) of titanium oxides on the surface of the metallic implant. Damage to that surface, resulting from wear and corrosion, leads to the release of titanium particles and ions into the surrounding tissues [16].

In our study, the presence of titanium particles in the periosteum of the maxilla and mandible was noticed in patients of both groups—the largest level was found in the mandibular periosteum in Group II. The obtained results may suggest the impact of wear processes in the jawbone fixations on the level of titanium particle concentration in the surrounding soft tissues, both during implant insertion and as a result of the fixed bone fragment micro-movements of fractured jaws or osteotomic segments of the mandible and maxillae. The observed higher concentration of titanium particles in the surrounding tissues sampled from the mandible in Group II patients may suggest a significant impact of the time criterion on the obtained research results. Compared to Group I, the prolonged presence of mini-plates and screws on the movable surface, i.e., the mandible, may involve a prolonged susceptibility to wear processes. Group I patients were treated with mostly double-plate bone fixations, which are associated with a more rigid stabilization of bone fragments, while Group II patients had single-plate fixations, thus they were less stiff, which may result in a higher intensity of micro-movements of the fixed osteotomic segments while healing and thereby cause fixation wear.

Moreover, in Group I, the mini-plates and screws that were removed from the mandibular body were localized not only in the region of the molars and the angle but also around the chin, premolars, and the canine, whereas in Group II patients, these fixations were localized only in the region of the molars and the angle of the mandible, i.e., at the site of attachments of strong masseter muscles to that bone. The contraction of the above muscles responsible for chewing movements may cause not only micro-movements of fixed bones, plates, and screws but also micro-movements of soft tissues covering the foreign body, which is the bone fixation, intensifying fretting–corrosion processes. All these abnormalities observed in both studied groups may contribute to the significantly different titanium concentrations observed in soft tissues surrounding mandible fixations in the studied Group I and II patients.

Bartucci et al. state that in cases of the loss of earlier implanted titanium prostheses of hip joints, the average concentration of titanium particles in the tissues surrounding the implant in patients may equal ca. 0.1% of the dry tissue mass [36]. Liu et al. proved higher levels of cobalt, chrome, and titanium in the blood and urine in periods longer than 3 years after the implantation. The highest levels of these metals were observed in ill patients where both the head and the acetabulum of the joint were made of metal—“metal-on-metal implants” [37]. The products of titanium degradation induce immunological reactivity by binding with blood serum proteins of high particle weight, particularly immunoglobulins, which may be the cause of skin and vessel inflammations, as well as allergic reactions [17]. Some authors also pay attention to the correlation between the presence of metallic particles originating from implants and the release of mediators of chronic inflammation and bone resorption. Connors et al. claim that the contact with the Ti6Al4V titanium alloy particles released from orthopedic prostheses of hip joints leads to the release of PGE_2_, IL-1, IL-6, and TNF-α, whereas there is no sign of cell necrosis in comparison to the phenomena resulting from the contact with Co-Cr particles [38]. The release of Ti6Al4V titanium alloy particles to the surrounding tissues compared to Co-Cr particles is potentially worse for prognosis as a higher number of visible macrophages activated in the study produce an elevated number of inflammatory mediators [39]. Similar phenomena may also occur for jaw fixations, where friction between screws and the miniplate occurs, as a result of corrosion in the tissue environment.

Both the presence of a titanium implant and the products of its wear and corrosion may lead to chronic inflammation around the inserted metallic body into the human body. This may cause an earlier loss of the implant (e.g., fixations, joint prostheses), which adversely affects the health of the treated patients and increases treatment costs [39,40].

The presence of metallic implants in the tissues of the human body and the products of their wear cause a range of reactions, which also lead to apoptosis. One of the key mediators of apoptosis is activated caspase-3, a cysteine protease enzyme responsible for activating cell endonucleases, leading to DNA fragmentation [41]. The lack of, or a deficiency in, this enzyme may cause many abnormalities in the composition of osseo-skeletal, muscular, immune, and haematopoietic systems. Caspase-3 also plays a vital role in rebuilding osseous tissues, affecting the apoptosis of bone marrow and osteoblasts [42].

This study focused on the significant rise in caspase-3 concentration in the periosteum covering jaw fixations. However, the concentration of this enzyme in the serum remained at a level of values characteristic of the control group. Caspase-3 concentration obtained in peri-implant tissues sampled from the mandible (Man I and Man II) indicates that the length of time these implants had been in the human body before their evacuation did not significantly change the concentration of the studied enzyme. Elevated caspase-3 concentration in the periosteum sampled from the maxilla in the Max II group of patients may stem from an indirect impact on the sampled tissues of the maxillary sinus environment and physiologically dominating processes of bone resorption on the anterior surface of the maxilla [43].

The products of degraded jaw fixations stimulate macrophages, fibroblasts, and T lymphocytes to produce pro-inflammatory cytokines such as TNF-α, IL-1, IL-6, metalloproteinases (MMPs), and other peptides. TNF-α is considered the key cytokine responsible for aseptic bone atrophy around metallic implants by way of intensifying inflammatory processes, inducing preosteoclast differentiation, increasing the pool of osteoclast precursors, promoting osteoclast survival, and increasing their activity. It also increases RANKL expression in osteoblasts and bone marrow cells [44,45].

Our research points to a statistically significant elevation of TNF-α concentration in peri-implant tissues in the maxilla. Still, in the blood serum, only a growing tendency in the concentration of this cytokine in Group I and II patients is observed. The increase in TNF-α concentration in the tissues removed from the maxilla (Max II) did not correspond to the concentration of this cytokine in the blood serum. The length of time the implant had been in the body had no impact on TNF-α stimulation within the tissues surrounding the mandible. Jamshidy et al. achieved similar outcomes when measuring TNF-α concentration in patients with an aseptic loss of hip joint prostheses. The cited authors stated that the patients’ age and sex, diagnosis, and the time period that had elapsed from the moment of prosthesis insertion until the onset of complications did not correlate with the level of evaluated cytokines [46].

TNF-α acts in a synergistic manner with IL-1α, IL-1β, and IL-6, inducing resorption of the osseous tissue. Granchi et al. assessed the concentration of TNF-α, IL-1β, and IL-6 in the bone serum in 35 patients with installed hip joint prostheses made of CrCoMo and TiAlV alloys, where an aseptic loss of these implants was observed. Unlike in our research, in 13 patients with installed hip joint prostheses made of titanium, they did not note changes in TNF-α concentration compared to the control group [47].

Xu et al. studied the impact of titanium nanoparticles on murine bone marrow cells. They observed a rise in gene expression responsible for the production of pro-inflammatory cytokines, including TNF-α. They also focused on the direct effect of the stimulation of osteoclastogenesis through titanium particles by RANKL [48]. Ramenzoni et al. evaluated the correlation between titanium nanoparticles and murine bone marrow macrophages. They observed increased macrophage differentiation in the direction of osteoclasts and intensified TNF-α production through macrophages under the influence of titanium particles. These authors suggest the possibility of inducing osteolytic processes around the titanium implants in bodies of mammals by titanium particles released from the implant directly through stimulating osteoclastogenesis, as well as stimulating pro-inflammatory reactions in macrophages (TNF-α) [49]. According to the authors, these processes may play a key role in aseptic pathogenesis and the osteolytic loss of implants inserted in human bodies. Our study of both groups concludes that there were no patients with an aseptic osteolytic loss of mini-plates and screws.

The studied patients in Groups I and II did not show increased TNF-α concentration in the sampled periosteum of the mandible (Man I and Man II) compared to the control group. The statistically significant rise in TNF-α and caspase-3 concentration in the tissues sampled from the maxilla in II Max group patients may result from the direct effect on the sampled tissues of the maxillary sinus environment through the former osteotomic fissure and a thin layer of mucosa and bone (manifold perforated by screws). Moreover, the anterior surface of the maxilla—the standard site of inserting mini-plates and screws immobilizing the osteotomic segments of the maxilla—is the dominant location for processes of bone resorption in the physiological sense. These may affect both TNF-α and caspase-3 concentration. The obtained minor increase in TNF-α and caspase-3 concentration in the periosteum sampled from the mandible of Group I and II patients may signify a slightly intensified chronic inflammatory condition and the rebuilding of extracellular matrix under the influence of cytokines released through activated macrophages in the presence of a foreign body—bone fixations and the products of their exploitation [50]. In our study, there was no connection between the concentration of titanium particles and the level of marked TNF-α and caspase-3 concentration in the tissues of Group I and II patients. Similar results were obtained by Theologie–Lidakis et al. [51], who examined morphological and chemical changes in retrieved titanium osteosynthesis plates and adjacent soft tissues.

Although implants are designed to immobilize spinal segments, small movements within the screws can cause abrasion of the metal, leading to the release of small titanium particles and their local deposition in the tissue. This has been confirmed not only by laboratory implant studies but also by inter-operative studies. Kaczmarek–Szczepańska and Polkowska discuss in their study the effect of titanium implants on the body, indicating that these levels may vary depending on individual factors, such as the patient’s metabolism and the time since surgery [52]. Specific values are provided by Nuevo–Ordóñez et al. They showed that patients with bone implants had increased levels of titanium in their blood compared to the control group. Before implantation, the titanium concentration was determined to be 1–2 ng/mL, while after implantation, it was already 10–30 ng/mL [53]. Similar results were obtained in the study by Ipacha et al., where the titanium concentration in patients after spine stabilization surgery increased proportionally to the number of screw and rod segments used, from 25 to even 50 ng/mL [54]. It should be emphasized that the above studies concern static implants. The issue of removable implants was addressed by Lukina et al. Their studies showed that the average concentration of titanium in the blood of patients with LSZ-4D implants was 85 ppb (range: 28–180 ppb), while in the control group, it was 30 ppb (range: 30–40 ppb). This means a 2.8-fold increase in the level of titanium in the blood compared to the control group. The concentration of aluminum (Al) did not show significant statistical differences, which may be due to the high variability of the results, while the concentration of vanadium (V) increased 4-fold, reaching 0.3 ppb compared to 0.08 ppb in the control group. The increased concentration of titanium and vanadium in the tissues clearly indicates high abrasion and dynamic degradation of the implant. Additionally, 5 of 25 patients (20%) experienced serious complications related to metallosis [55].

In the study conducted by the authors of this article, the concentration of titanium in both the periosteum and blood serum was lower compared to the results of Nuevo–Ordóñez et al. [53], Ipacha et al. [54], and Lukina et al. [55] However, it should be considered that the patients of the USK in Bialystok were young people whose bone fixations were removed before 1 year, and in some cases after only 4 months.

## 4. Materials and Methods

### 4.1. Ethical Aspects

The research was conducted in accordance with the Declaration of Helsinki—1964 (with modification October 2013, Fortaleza, Brazil). All patients were informed about the manner of this study and the collection of research material (blood and periosteum) and agreed in writing to participate in the experiment. The protocol of this study was approved by the Bioethics Committee of the Medical University of Bialystok (R-I-002/514/2013—24 October 2013, APK.002.71.2023—19 January 2023, and APK.002.72.2023—19 January 2023; from 24 October 2013, until 30 December 2025).

### 4.2. Study Group

**Figure 9:**
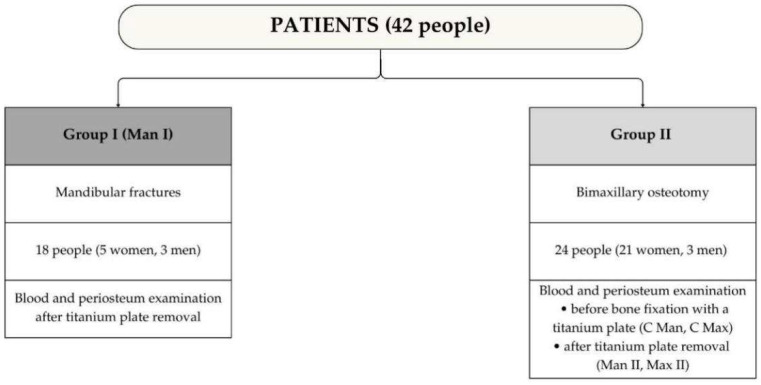
Characteristics of study groups.

The study involved 42 patients from the Department of Maxillofacial and Plastic Surgery of the Medical University of Bialystok. The participants were divided into two research groups (Figure 9). Group I included patients hospitalized due to isolated mandibular fractures (Man I). In these patients, tissue trauma-induced inflammation developed before admission to the hospital. Therefore, preoperative determination of caspase-3 and TNF-α concentrations in blood and periosteum was not performed. Group II included patients qualified for orthognathic surgery (bimaxillary osteotomy) for indications related to dentofacial deformities. In this group, both the mandible (Man II) and the maxilla (Max II) were operated on. These patients also constituted the control group (C Man, C Max) because they were in good general condition before the surgical intervention, and their periosteum remained intact. The lack of inflammation eliminated the influence of inflammatory factors on the concentration of caspase-3 and TNF-α before surgery. Biological material (blood and periosteum) collected from these patients before surgery was used as a comparative (control) material. The results of these patients before surgery were compared with the results after surgery. Additionally, the results of Group II patients (Man II, Max II) were compared with the results of Group I patients (Man I).

Group I (Man I) consisted of 18 patients (5 women and 13 men) aged 20–29 years (mean age—22 years and 3 months). The most common causes of injuries in this group were physical assaults (83.3% of patients), accidents (11.1%), and injuries resulting from physical activity (5.6%). Group II (Man II, Max II, and, simultaneously, the control group C Man, C Max) consisted of 24 patients (21 women and 3 men aged 20–29, with mean age of 23 years and 11 months).

The criteria for qualifying patients for the study were: age of 20–30 (at this stage of life, a human organism reaches its peak bone mass, and the processes of build-up and resorption of bone tissue are balanced; such a selection of patients, as confirmed by other authors, significantly minimized the impact on the obtained results of metabolic processes observed in the bone in other age ranges), presence of maxillary bone fixations after fracture treatment or dentofacial deformities, a non-inflammation-induced healing process starting from inserting fixations until their removal, no previous treatment of bone fractures or osteotomies performed, no inflammatory conditions and metabolic or systemic diseases confirmed by interview, subjective and additional tests, treatments free of hormone, anticoagulant, anticonvulsant, diuretic medications, magnesium preparations or vitamin D before the injury/osteotomy, declared abstinence from alcohol or intoxicating drugs during the previous 2 months, and no active foci of odontogenic infections.

The study group of patients was limited to those who underwent both bone fixation and titanium plate removal at the Department of Maxillofacial and Plastic Surgery of the Medical University of Bialystok. The most common reason for fixation removal (26 patients) was the patient’s request to remove the implants after clinical healing of a fracture or osteotomy, followed by discomfort associated with palpable fixations (8 patients), increased sensitivity to cold (7 patients), and periodic local redness of the facial skin caused by changes in ambient temperature (1 patient). Patients who experienced postoperative complications were excluded from the study.

### 4.3. Surgical Procedure

Evaluation of bone union was conducted through regular radiographic imaging and confirmed intraoperatively. Radiological assessments comprised teleroentgenogram in the PA (Posterior–Anterior) projection and cephalometry in the lateral projection (Cranex 3D, Soredex, Tuusula, Finland). The titanium bone fixations (ChM Lewickie Sp. z o. o., Lewickie, Poland) were removed under general anesthesia; all surgical interventions were conducted under general anesthesia by the consistent surgical team (J.B.). In patients in whom bone fusion occurred, during the procedure of bone fixation removal (miniplate and screw), fragments of the periosteum adjacent to the titanium Ti6Al4V mini-plates were collected and cut out as standard during the removal of these fixations. In patients in Group I (with mandibular fractures), the titanium fixation-removal procedure was performed after 4–10 months (average of approximately 6 months), while in Group II (with dentofacial deformities), it was performed after 11–12 months (average of approximately 11–12 months). Before the procedure, 10 mL of blood (fasting) were collected from each of these patients for standard biochemical tests, and the serum was stored at −80 °C for further tests. The biochemical and morphological parameters of the blood of the examined patients met the standards and were not included in this article. In the control group, C—blood serum and small fragments of the periosteum were collected during bimaxillary osteotomy before titanium mini-plates and screws were inserted.

### 4.4. Preparation of Tissue Homogenates

The removed tissues were immediately frozen in liquid nitrogen and stored at −80 °C until use. To prepare the material for the study, the tissue samples were rinsed in ice-cold PBS (0.02 mol L^−1^, pH 7.0–7.2) to clear any remaining blood components. They were then minced into small pieces and homogenized, then diluted in PBS using a homogenizer on ice (Omni TH, Omni International, Kennesaw, GA, USA) and treated with an ultrasonic cell disrupter (1800 J per sample, 20 s × 3 on ice, UP 400S; Hielscher, Teltow, Germany) for further cell membrane breakdown. Homogenates were centrifuged for 5 min at 5000× *g*. The supernatant was then removed and either assayed immediately or divided into aliquots and stored at −80 °C.

### 4.5. Biochemical Analysis

The levels of TNF-α and caspase-3 in serum and tissue homogenates were measured using ELISA kits in accordance with the manufacturers’ instructions (Human TNF-alpha Quantikine ELISA Kit; R&D Systems, Minneapolis, MN, USA and ELISA Kit for Human CASP-3; Wuhan EIAAB SCIENCE Co., Ltd, Wuhan, China).

### 4.6. Titanium Concentration Analysis

The concentration of titanium ions in the tissues was measured using a Hitachi S-3000N scanning electron microscope (Hitachi, Tokyo, Janan) with an X-ray microanalyzer, equipped with a freezing stage for testing biological preparations and determining the chemical composition (EDS Ultra Dry control microanalysis attachment Thermo Fisher Scientific, Waltham, MA, USA). The use of the freezing stage allowed for the observation of wet samples without the need for drying and vapor deposition. The preparations on the freezing stage were tested at a temperature of −20 °C.

### 4.7. Statistical Analysis

Statistical comparisons were performed by a two-way analysis of variance followed by the Newman–Keuls test. If variances were heterogeneous among groups, the Mann–Whitney test was used instead. The correlation between the concentrations of titanium, TNF-α, and caspase-3 was calculated using the Spearman correlation coefficient (a nonparametric rank test). *p* < 0.05 was considered statistically significant.

## 5. Conclusions

In Group II patients (Max II), an increase in the concentration of TNF-α and caspase-3 was observed in homogenized tissues, which may indicate that maxillary bone fixations in patients treated for dentofacial deformities may cause chronic inflammatory processes and increased apoptosis. The duration from the placement of titanium fixations to their removal, the number of mini-plates and screws applied, and the type of surgery performed (osteosynthesis after a fracture or osteosynthesis after an osteotomy) have a significant impact on the concentration of titanium particles in the tissues surrounding the mandibular fixations. However, further studies are needed to confirm these findings.

The finding in our study of ongoing chronic inflammation around mini-plates and screws and their wear products may indicate the need to remove the maxillary fixation after the bone union of fractured or osteotomized jaws. The mechanism of these results requires further research. The authors plan further studies to assess the effect of fixations connecting the maxillary bones and the resulting by-products on the induction of chronic inflammation and its effect on the surrounding tissues.

## Data Availability

The raw data supporting the conclusions of this article will be made available by the authors without undue reservation.
